# From Time‐Limited Therapy to Treatment‐Free Observation: The Evolving Role of MRD in CLL Management

**DOI:** 10.1111/ejh.70200

**Published:** 2026-04-19

**Authors:** Enrica Antonia Martino, Santino Caserta, Ernesto Vigna, Antonella Bruzzese, Maria Eugenia Alvaro, Nicola Amodio, Eugenio Lucia, Virginia Olivito, Caterina Labanca, Francesco Mendicino, Mamdouh Skafi, Valter Gattei, Fortunato Morabito, Massimo Gentile

**Affiliations:** ^1^ Hematology Unit, Department of Onco‐Hematology AO of Cosenza Cosenza Italy; ^2^ Department of Experimental and Clinical Medicine University of Catanzaro Catanzaro Italy; ^3^ Emergency and Internal Medicine Department Saint Joseph Hospital East Jerusalem Palestine; ^4^ Clinical and Experimental Onco‐Hematology Unit Centro di Riferimento Oncologico di Aviano (CRO) IRCCS Aviano Italy; ^5^ AIL Sezione di Cosenza Cosenza Italy; ^6^ Department of Pharmacy, Health and Nutritional Science University of Calabria Rende Italy

**Keywords:** artificial intelligence, Bruton tyrosine kinase inhibitors, chronic lymphocytic leukemia, circulating tumor DNA, minimal residual disease, treatment‐free observation, undetectable MRD, venetoclax

## Abstract

The integration of measurable residual disease (MRD) into the management of chronic lymphocytic leukemia (CLL) has emerged as a major advance in risk stratification and trial design, particularly in the context of time‐limited, targeted regimens. High‐sensitivity MRD assessment, enabled by multicolor flow cytometry, allele‐specific oligonucleotide PCR, and next‐generation sequencing (NGS), provides a robust, quantifiable measure for depth of remission and long‐term outcomes. Landmark trials—including CLL14, MURANO, CAPTIVATE, and GLOW—have consistently demonstrated that achieving undetectable MRD (uMRD) strongly predicts prolonged progression‐free survival (PFS) and overall survival (OS), within a range of chemoimmunotherapy and venetoclax‐based time‐limited regimens. In selected clinical trials, MRD assessment has been prospectively incorporated into strategies exploring time‐limited therapy and MRD‐adapted discontinuation, although routine MRD‐guided decision‐making in clinical practice remains investigational. CLL research is increasingly focused on treatment‐free observation in select patients achieving deep and sustained remissions, with MRD playing a central prognostic role. Emerging technologies, including circulating tumor DNA (ctDNA) monitoring and artificial intelligence (AI)‐driven predictive modeling, promise to further refine risk stratification and personalize therapy. This review summarizes the current evidence supporting MRD as a prognostic biomarker and clinical trial endpoint, discusses investigational MRD‐adapted strategies, and outlines future directions—including ctDNA and AI‐based tools—that may ultimately support more individualized treatment duration and treatment‐free observation in CLL.

## 1 Introduction

Over the past two decades, the treatment of chronic lymphocytic leukemia (CLL) has evolved dramatically—from cytotoxic chemoimmunotherapy (CIT) to targeted, precision‐based approaches. Historically, frontline regimens such as fludarabine‐cyclophosphamide‐rituximab (FCR) or bendamustine‐rituximab (BR) achieved durable responses mainly in younger, fit patients with mutated *IGHV* and intact *TP53* [1, 2]. However, despite initial efficacy, most patients eventually experience relapse of the disease, underscoring the limitations of CIT in providing sustained remissions or a cure [3].

The introduction of novel oral agents targeting B‐cell receptor (BCR) signaling and apoptotic pathways has dramatically improved outcomes and shifted CLL therapy toward individualized, biology‐driven management. Bruton tyrosine kinase inhibitors (BTKis) such as ibrutinib [4, 5, 6], acalabrutinib [7, 8], zanubrutinib [9, 10, 11, 12, 13] and the non‐covalent BTKi pirtobrutinib [14], along with the Bcl‐2 inhibitor venetoclax [15, 16, 17], have reshaped the therapeutic paradigm. These agents achieve durable remissions with greater tolerability than chemotherapy, offering effective disease control even in high‐risk subgroups, including patients with unmutated *IGHV* or *TP53* abnormalities [18].

However, the expanding arsenal of targeted therapies has raised key questions about optimal treatment duration and the feasibility of safely discontinuing therapy without early disease recurrence. Originally developed as continuous, indefinite treatments—particularly BTK inhibitors—these agents have since paved the way for time‐limited regimens that leverage their complementary mechanisms of action. Fixed‐duration combinations, such as venetoclax plus an anti‐CD20 antibody (e.g., venetoclax–obinutuzumab in the CLL14 trial) or venetoclax combined with BTK inhibitors (e.g., ibrutinib or acalabrutinib), have redefined treatment goals by enabling time‐limited therapy and achieving high rates of deep molecular remissions within predefined treatment intervals, irrespective of MRD status at treatment discontinuation in clinical trials and selected approved regimens [19, 20, 21, 22].

Selecting the most appropriate BTK inhibitor to combine with venetoclax requires a patient‐centered approach that carefully considers efficacy alongside the safety profile of each agent. Differences in the biological activity and tolerability of individual BTK inhibitors may influence their use within continuous or fixed‐duration treatment strategies and should be taken into account when tailoring therapy in clinical practice [23]. In this context, compared with acalabrutinib, ibrutinib may induce a more pronounced mobilization of quiescent CLL cells from lymphoid niches, potentially contributing to a “compartment shift” toward the peripheral blood (PB). This process is accompanied by a higher induction of the pro‐apoptotic protein BIM, which saturates mitochondrial Bcl‐2 and renders the remaining unbound Bcl‐2 more critical for cell survival [23, 24, 25]. As a result, venetoclax may encounter a larger pool of primed, apoptosis‐sensitive cells, enhancing its cytotoxic effect.

The role of fixed‐duration regimens—particularly those combining BTK and BCL2 inhibition—is rapidly evolving and may soon represent a dominant treatment paradigm in defined patient subsets [21]. Ongoing randomized trials comparing venetoclax–obinutuzumab with various BTKi–venetoclax combinations (NCT04608318, NCT05057494, NCT06073821) will be key to determining the optimal balance between efficacy, safety, and duration of therapy. Recently, results from the phase III CLL17 study have shown no significant differences in efficacy outcomes among the compared treatment strategies, suggesting that the optimal sequencing or combination of venetoclax–obinutuzumab and BTKi–venetoclax regimens remains to be defined [26].

Within this evolving framework, new combination regimens are marking a decisive shift from simple disease suppression toward the goal of achieving deeper and more durable remissions, with treatment duration increasingly guided by the biological depth of remission rather than dictated by fixed timeframes. Minimal/measurable residual disease (MRD) denotes the amount of malignant cells that remain present at low levels in patients undergoing therapy [27]. The persistence of resistant clones is favored by the microenvironment, which provides pro‐survival signals [28]. The interaction between malignant B cells and their surrounding milieu, which encompasses myeloid cells, mesenchymal stromal cells, and immune effectors such as T and NK cells, promotes CLL cell survival and expansion, immune escape, and contributes to therapeutic resistance [29]. A recent study reveals that CLL cells can reside within bone marrow (BM) fibroblasts, evading cytotoxic and immune clearance. This protective niche supports dormant, therapy‐resistant clones through stromal‐derived survival signals, explaining relapse despite deep molecular responses. These insights highlight the tissue (BM and/or lymph node) microenvironment as a key therapeutic target. Strategies aimed at disrupting fibroblast–leukemia interactions, including inhibition of the CXCR4/CXCL12 axis, blockade of adhesion molecules (e.g., VLA‐4, CD44), or interference with PI3K and NF‐κB signaling, could sensitize sheltered cells to apoptosis. In this context, it is noteworthy that high VLA‐4 expression has prognostic significance in CLL during BTKi therapy [30, 31, 32, 33] and that resistance‐conferring BTK mutations preferentially emerge under ibrutinib treatment within the CXCR4dim/CD5bright CLL subpopulation [34], which represents recently egressed tissue‐derived cells. Combining niche‐disrupting agents with BCL2 or BTK inhibitors, or integrating advanced immunotherapies like bispecific antibodies or CAR‐T cells, may enhance MRD clearance. Ultimately, dismantling this fibroblast‐mediated sanctuary represents a crucial step toward converting transient deep remissions into durable, treatment‐free disease control.

The assessment of MRD—through high‐sensitivity flow cytometry, allele‐specific PCR, or next‐generation sequencing (NGS)—has become a useful tool for quantifying response, predicting relapse risk, and identifying candidates for time‐limited treatment followed by treatment‐free observation (TFO) in selected settings [35, 36].

Recent developments have further expanded this paradigm. The integration of circulating tumor DNA (ctDNA) analysis allows detection of residual disease beyond traditional MRD assays, potentially capturing recurrence earlier than flow cytometry [37]. Meanwhile, artificial intelligence (AI)–based predictive modeling—integrating MRD kinetics, mutational profiles, and immune recovery—holds promise for personalized therapy optimization, enabling more refined, potentially adaptive treatment algorithms in the future [38].

In this review, we summarize the evolution of therapeutic approaches in CLL, emphasizing how MRD is beginning to influence clinical decision‐making and trial design. We discuss biological and clinical predictors of durable MRD negativity, examine how novel technologies such as ctDNA analysis and AI‐based modeling might refine response assessment in the future, and outline the practical and conceptual implications of incorporating MRD into both research and evolving CLL management.

## 2 Assessment of MRD in CLL

MRD provides a highly sensitive quantification of leukemic burden beyond the resolution of conventional morphology. The degree of MRD clearance closely reflects the depth of therapeutic response and has consistently been associated with clinical outcomes. As treatment paradigms have evolved—from chemoimmunotherapy to fixed‐duration targeted regimens—MRD assessment has emerged as a robust biomarker for response evaluation and risk stratification, while its role in guiding treatment decisions in routine clinical practice remains under investigation [39].

By capturing subclinical disease persistence, MRD offers a biological link between apparent clinical remission and long‐term disease control, supporting a more individualized approach to patient management. However, its broader implementation in routine care still requires further harmonization of methodologies, prospective validation, and integration into standardized treatment algorithms and clinical guidelines.

Currently, three main analytical platforms are used for MRD detection in CLL: multicolor flow cytometry (MFC), allele‐specific oligonucleotide or digital droplet polymerase chain reaction (ASO‐qPCR/ddPCR), and high‐throughput sequencing (HTS or NGS). These methods differ in terms of analytical sensitivity, standardization, cost, and applicability across clinical and research settings [40].

### 2.1 MFC

MFC remains the foundational method for MRD detection and underpins the recommendations of the European Research Initiative on CLL (ERIC). The technique identifies residual clonal B cells co‐expressing CD19 and CD5, often associated with aberrant expression of CD20, CD43, CD79b, and CD81. With optimized antibody panels and standardized gating strategies, MFC achieves a typical sensitivity of 10^−4^ and, in specialized settings, may reach 10^−5^ [35, 40].

The main advantages of MFC include rapid turnaround time, relatively low cost, and wide availability, making it the most widely used method for MRD assessment in routine clinical practice. Standardization efforts by ERIC and the European Society for Clinical Cell Analysis (ESCCA) have significantly improved reproducibility and inter‐laboratory comparability.

However, the technique requires fresh, viable samples and experienced technical interpretation. This is particularly relevant in the post‐treatment setting, where regenerating normal B cells may phenotypically resemble residual leukemic cells, potentially complicating analysis. Despite these limitations, MFC remains the practical “workhorse” for MRD evaluation, offering an optimal balance between sensitivity, feasibility, and accessibility in most clinical settings.

### 2.2 Molecular Methods: ASO‐qPCR and ddPCR

Molecular assays such as ASO‐qPCR and ddPCR detect patient‐specific immunoglobulin heavy chain (IGH) rearrangements defined at diagnosis. These techniques offer superior analytical precision and reproducibility, reaching sensitivities of 10^−5^. ddPCR, in particular, enables absolute quantification without calibration curves, improving cross‐center consistency. Their major limitation lies in the need for pretreatment DNA to design individualized primers, which may not always be available. Furthermore, PCR‐based methods require specialized laboratory infrastructure and are more resource‐intensive than flow cytometry [35, 36, 40].

Nevertheless, ASO‐qPCR and ddPCR have provided the methodological backbone for MRD quantification in pivotal clinical trials—such as CLL8, MURANO, and CLL14—establishing key benchmarks for correlation between MRD depth and long‐term outcomes.

### 2.3 Molecular Methods: NGS

NGS represents the most advanced evolution of MRD assessment, offering unparalleled sensitivity and molecular resolution. Once the baseline clonotype is established, leukemic DNA can be detected at levels as low as one malignant cell per one million normal leukocytes (10^−6^)—a sensitivity that surpasses both MFC (10^−4^–10^−5^) and ASO‐PCR (10^−5^) [35, 36, 40].

Unlike ASO‐PCR, NGS does not require individualized primers if standardized immunoglobulin libraries are used. The clonoSEQ assay (Adaptive Biotechnologies, Seattle, WA) is FDA‐cleared for MRD quantification and provides reproducible results suitable for clinical trials. Use of unique molecular identifiers (UMIs) and calibration controls minimizes amplification bias and sequencing artifacts, ensuring quantitative accuracy and harmonization across institutions.

Beyond quantification, a separated assessment through NGS often requiring a process of purification of neoplastic cells may also offer an additional dimension by capturing clonal evolution and subclonal diversification—key processes driving therapeutic resistance and relapse under targeted agents such as BTK or BCL2 inhibitors. Thus, NGS not only measures residual burden but also reveals the biological complexity of disease persistence.

Despite these strengths, NGS is limited by cost, longer turnaround time, and the need for bioinformatics expertise. It also requires pretreatment DNA to define the index clonotype and may detect low‐frequency rearrangements of uncertain clinical significance, complicating interpretation in long‐term remission. Nonetheless, due to its reproducibility and scalability, in the near future NGS may represent the reference standard for MRD quantification in research and regulatory settings [41], especially for trials incorporating MRD‐guided treatment discontinuation or retreatment strategies in carefully defined contexts.

### 2.4 Choosing the Appropriate MRD Platform

In clinical practice, the choice of MRD assay should be guided by its intended use and available infrastructure. MFC remains appropriate for routine post‐treatment monitoring, owing to its accessibility and rapid turnaround time. In contrast, molecular and sequencing‐based methods are more commonly employed in clinical trials, for regulatory endpoints, or in research settings requiring higher analytical sensitivity. PB MFC (with sensitivity up to 10^−5^) shows good concordance with BM MRD assessed by PCR or NGS, typically at a sensitivity of 10^−4^, supporting the use of less invasive sampling strategies in many clinical contexts. This correlation has facilitated the incorporation of MRD assessment into several clinical trials—such as CLL14, CAPTIVATE, and BOVen—where MRD has been used primarily as a measure of depth of response and as a surrogate endpoint. However, it should be noted that not all these studies employed MRD to guide treatment discontinuation. In particular, CLL14 was based on a fixed‐duration treatment approach rather than an MRD‐driven strategy. Supporting these observations, Raponi et al. [42] performed a comparative analysis of MFC and ASO IgH RQ‐PCR in paired BM and PB samples from patients treated with chemo‐immunotherapy. The study demonstrated a high concordance between MFC and ASO‐PCR, confirming the reliability of both techniques for MRD assessment. Notably, ASO‐PCR showed higher analytical sensitivity, detecting residual disease at levels as low as 10^−5^–10^−6^ in a subset of samples that were MRD‐negative by MFC.

### 2.5 Emerging Technology: Hairpin Adapter–Mediated Amplification PCR (HAT‐PCR)

A recent review introduces the Hairpin Adapter–mediated Amplification Technique PCR (HAT‐PCR) as a highly sensitive and practical molecular tool for detecting MRD in CLL and multiple myeloma [43]. As described earlier, traditional MRD assays such as MFC, ASO‐PCR, and NGS achieve sensitivities between 10^−4^ and 10^−6^ but are often limited by complexity, cost, and the need for patient‐specific reagents. HAT‐PCR overcomes these constraints. Baseline IGH rearrangements were identified using NGS, either through the commercially available LymphoTrack assay or via a custom‐developed two‐step PCR library preparation approach. This strategy enabled successful IGH sequencing in all analyzed CLL BM samples (37/37). MRD quantification using HAT‐PCR was performed in 125 BM or PB specimens from 36 CLL patients, with two samples showing MRD levels below 10^−6^. Comparative analysis of paired samples demonstrated that HAT‐PCR provided significantly greater sensitivity than MFC. Importantly, the assay demonstrated robust performance with low DNA input per individual reaction (as little as 50 ng). While this supports technical feasibility in routine and retrospective settings, a limit of detection of 10^−6^ requires analysis of a sufficiently large total number of cell equivalents, achieved by cumulative DNA input across multiple reactions.

The assay's simplicity and robustness make it particularly suited for integration into clinical laboratories. Compared to NGS, it provides similar sensitivity with faster processing and lower cost while maintaining digital precision through UMIs.

Although promising as a technique of MRD quantification, HAT‐PCR has not yet reached the level of independent clinical validation in CLL that would support its use as a standard prognostic tool or to drive treatment decisions. If validated across centers, it could facilitate widespread MRD‐guided therapy, enabling personalized treatment duration and advancing the goal of treatment‐free remission. At present, however, its use should be considered exploratory and confined to specialized or research settings.

Ultimately, the integration of these technologies into a unified, standardized workflow will be crucial for the widespread implementation of MRD‐informed approaches to CLL management. Table 1 summarizes the main characteristics of the three methodologies.

**TABLE 1:** Comparison of MRD detection technologies in CLL.

Feature	Flow cytometry (MFC)	ASO‐PCR/ddPCR	Next‐generation sequencing (NGS)	HAT‐PCR
Principle	Immunophenotypic detection of residual CLL cells through aberrant CD marker expression	Patient‐specific amplification of IGH rearrangements (allele‐specific)	Sequencing of IGH/IGK/IGL clonotypes to identify and track the dominant leukemic clone	Hairpin Adapter–mediated PCR using universal primers and unique molecular identifiers (UMIs)
Sensitivity (limit of detection)	10^−4^ to 10^−5^	10^−5^	10^−6^	10^−6^
Quantitative accuracy	Moderate – semi‐quantitative; depends on gating and operator expertise	High – absolute quantification (ddPCR); standard curves required for ASO‐PCR	Very high – standardized calibration enabling detection of minor subclones	High – digital quantification via UMIs with minimal amplification bias
Sample requirement	Fresh, viable cells from peripheral blood or bone marrow	Pretreatment DNA for primer design and follow‐up DNA for monitoring	Pretreatment DNA is required to identify the clonotype	50–100 ng DNA from peripheral blood or bone marrow
Turnaround time	Same‐day (hours)	2–3 days	5–7 days	24–48 h
Need for patient‐specific reagents	No	Yes (custom primers/probes)	No (standardized immunoglobulin libraries)	Yes (custom primers/probes)
Detection of clonal evolution	No	Limited	Yes (tracks evolving subclones and mutations)	Detects co‐existing IGH rearrangements present at baseline
Cost	Low	Moderate–high	High	Moderate (lower than NGS, higher than MFC)
Infrastructure requirements	Flow cytometer and trained operator	Specialized PCR setup with quality controls	Advanced sequencing facilities and bioinformatics support	Standard molecular laboratory; no NGS platform required
Reproducibility/standardization	High (ERIC‐standardized panels)	Moderate; variable across laboratories	Very high; standardized (e.g., clonoSEQ)	High potential for inter‐laboratory harmonization
Clinical applicability	Routine hospital use: cornerstone for clinical monitoring	Widely used in clinical trials; limited scalability	Gold standard for research and regulatory endpoints	Broad; suitable for real‐time MRD monitoring and clinical use
Main advantages	Widely available, rapid, low cost; standardized (ERIC protocol)	Quantitative and reproducible; established benchmark in clinical trials	Deepest sensitivity; detects clonal evolution; harmonized data	Combines NGS‐level sensitivity with PCR simplicity, low DNA input, and cost‐effective
Main limitations	Lower sensitivity; needs fresh samples; operator‐dependent	Requires patient‐specific setup; time‐consuming	Expensive; longer turnaround; complex data analysis	Requires multicenter validation; emerging technology
Best use scenario	Routine clinical follow‐up and hospital‐based MRD monitoring	Reference tool in legacy clinical trials	Regulatory‐grade trials and research on resistance or relapse	Real‐time MRD‐guided therapy; cost‐efficient molecular monitoring

### 2.6 Standardization and Harmonization

Accurate and reproducible MRD assessment depends critically on rigorous standardization of laboratory techniques and reporting practices. Recognizing MRD's growing role as both a prognostic biomarker and a potential therapeutic guide, the European Research Initiative on CLL (ERIC), in partnership with the European Society for Clinical Cell Analysis (ESCCA), has developed comprehensive harmonized guidelines for MRD detection in CLL [35]. These guidelines encompass key aspects of assay performance, including sample handling, antibody panel configuration, instrument setup, data acquisition, and analytical interpretation.

One of the major achievements of these harmonization efforts has been the establishment of universally accepted thresholds for MRD quantification. Specifically, MRD negativity (uMRD) is defined as fewer than 0.01% leukemic cells, corresponding to a sensitivity of 10^−4^, or one CLL cell among 10 000 leukocytes. This standardized definition ensures consistent interpretation across laboratories and clinical trials, enabling meaningful comparison of patient outcomes and facilitating pooled analyses in meta‐studies.

Standardization has become particularly crucial in the era of MRD‐informed, time‐limited therapy, especially within clinical trials. Variability in assay sensitivity or reporting can significantly affect clinical decisions, such as determining whether to discontinue targeted therapy or resume treatment. Harmonized reporting thus underpins the safe and effective integration of MRD into future clinical algorithms and is vital for regulatory recognition, as agencies such as the FDA and EMA increasingly consider MRD a surrogate endpoint in therapeutic trials.

Table 2 summarizes some recommendations and the clinical relevance of MRD in CLL [27, 35].

**TABLE 2:** Proposed standardization and reporting of MRD in CLL.

Aspect	Recommendation/standard	Clinical or research relevance
Mrd threshold	uMRD defined as < 0.01% leukemic cells (1 CLL cell in 10 000 leukocytes)	Enables consistent reporting and comparison across trials; guides clinical decisions on therapy discontinuation
Sample type	Peripheral blood preferred for routine monitoring; bone marrow for high‐risk or research scenarios	Standardizes sampling and correlates PB versus BM MRD; allows longitudinal monitoring
Assay methodology	Flow cytometry (MFC) as primary method; molecular methods (ASO‐PCR, NGS) as adjunct for research/deep MRD	Harmonizes technique selection; aligns clinical versus trial‐based sensitivity
Panel composition (MFC)	6–8 color antibody panels including CD19, CD5, CD20, CD43, CD79b	Ensures accurate identification of CLL cells and minimizes false positives
Data acquisition	Minimum of 500 000–1 000 000 events per sample recommended	Improves the sensitivity and reliability of MRD quantification
Reporting requirements	Include sample type, assay method, number of events acquired, limit of detection/quantification, and MRD result (% and absolute counts)	Facilitates reproducibility, accurate interpretation, and clinical translation
Clinical application	In selected clinical trials, MRD negativity has been used to inform therapy discontinuation or observation, whereas detectable MRD can guide retreatment planning; outside these settings MRD is primarily a prognostic biomarker	Integrates MRD into personalized, time‐limited treatment strategies
Quality assurance	Participation in external QA programs (ERIC, EuroFlow) is recommended	Ensures inter‐laboratory consistency and compliance with guidelines

In summary, each MRD detection platform offers unique contributions to disease monitoring in CLL. MFC provides a rapid, accessible, and standardized method suitable for routine clinical use; ASO‐PCR and ddPCR deliver high‐precision quantification essential for clinical trial validation; and NGS stands at the forefront of translational research, combining maximal sensitivity with insight into disease biology. Emerging technologies such as HAT‐PCR may soon bridge the gap between analytical sensitivity and clinical practicality. The future development of MRD‐driven approaches in CLL will likely benefit from improved integration of available methodologies within more standardized workflows. Efforts toward harmonized protocols, greater cross‐laboratory comparability, and enhanced data integration may facilitate a more consistent use of MRD as a prognostic marker and potential surrogate endpoint. In this context, MRD assessment could increasingly support clinical decision‐making, including considerations around treatment duration and retreatment, although its role in routine practice continues to evolve and is currently best established within prospective clinical trials rather than unselected real‐world use.

## 3 Prognostic Significance of MRD in CLL

The prognostic significance of measurable MRD in CLL has been established across diverse therapeutic settings. Achieving uMRD is consistently associated with prolonged progression‐free and overall survival; however, its role as a surrogate endpoint may vary depending on the treatment strategy. In particular, while MRD retains strong prognostic value, it may not reliably function as a surrogate marker of outcome in the context of continuous BTK inhibitor therapy, underscoring the need to interpret MRD results in light of the specific treatment modality and clinical context. Moreover, while uMRD has consistently demonstrated strong prognostic value in chemoimmunotherapy and venetoclax–anti‐CD20 regimens, its role as a predictor of progression‐free and overall survival appears less clearly defined in the context of BTK inhibitor–based therapies and fixed‐duration BTKi–venetoclax combinations.

### 3.1 MRD as a Prognostic Marker in Chemoimmunotherapy

During the chemoimmunotherapy era, the German CLL Study Group (GCLLSG) demonstrated that uMRD after fludarabine, cyclophosphamide, and rituximab (FCR) strongly correlated with prolonged PFS and OS. In a pooled analysis of over 2300 patients, those achieving uMRD (< 10^−4^) had a median PFS exceeding 10 years versus ~4 years for MRD‐positive cases [44]. This prognostic impact was shown to be largely independent of IGHV mutation status and cytogenetic risk [45], supporting MRD negativity as a robust marker of favorable outcome across biological subgroups. However, a more nuanced interpretation is required. While within each biological subgroup patients achieving uMRD consistently experience superior outcomes compared with MRD‐positive cases, the absolute progression‐free survival (PFS) still varies according to baseline disease biology. In particular, patients with TP53 disruption or unmutated IGHV, even when achieving uMRD, generally have shorter PFS compared with biologically favorable subsets. Therefore, MRD status should be interpreted as a strong, but not fully uniform, prognostic marker, reflecting depth of response within a given biological context rather than completely overriding the underlying disease risk profile.

High‐sensitivity flow cytometry and NGS refined MRD assessment [46]. The FILO study (> 500 FCR‐treated patients) showed that six‐ and eight‐color flow cytometry with detection limits of 0.7 × 10^−5^ identified prognostic value even below 0.01%. PB MRD closely mirrored BM results, confirming flow cytometry as a practical alternative to invasive sampling. NGS further improved sensitivity (10^−6^), detecting residual disease missed by flow cytometry and linking true molecular negativity to longer remissions. These methods are complementary: flow cytometry is feasible for routine use, while NGS provides deeper biological precision.

### 3.2 MRD as a Prognostic Marker in Continuous Targeted Therapy

Continuous BTK inhibition has fundamentally changed CLL management, demonstrating that long‐term disease control can be maintained even without deep molecular remission. The RESONATE‐2 study [47] evaluated first‐line ibrutinib in older patients and confirmed exceptional long‐term PFS (median 8.9 years) compared with chlorambucil (1.3 years). Importantly, MRD negativity rates were not reported, consistent with the predominantly cytostatic mechanism of BTK inhibition, which constrains leukemic proliferation rather than inducing deep molecular clearance. Nevertheless, continuous ibrutinib therapy yielded durable remissions, indicating that the presence of low‐level residual disease does not preclude sustained disease control. These observations suggest that, although MRD serves as a robust prognostic marker in the context of chemoimmunotherapy, its relevance may be attenuated with continuous BTK inhibition.

Similarly, the final results of the ALPINE trial [9], which compared continuous therapy with the second‐generation BTK inhibitor zanubrutinib versus ibrutinib in relapsed/refractory CLL, further illustrate this treatment paradigm. Zanubrutinib demonstrated superior tolerability, higher overall response rates, and prolonged PFS, emphasizing the need to contextualize MRD findings according to the therapeutic mechanism. Accordingly, interpretation of MRD findings should be contextualized within the specific treatment paradigm, acknowledging that the depth of molecular remission does not invariably translate into sustained clinical benefit under continuous targeted therapy.

The ELEVATE‐TN study, which evaluated frontline acalabrutinib with or without obinutuzumab versus chlorambucil–obinutuzumab, showed that the addition of obinutuzumab to acalabrutinib significantly increased uMRD rates (40.9% vs. 8.8% with acalabrutinib monotherapy and 8.3% with chlorambucil–obinutuzumab), as assessed by flow cytometry (sensitivity 10^−4^) in both PB and BM [7]. However, MRD‐stratified PFS outcomes were not reported, and therefore the study does not establish whether achieving uMRD translated into improved PFS in this setting. This distinction is important, as it does not negate the prognostic value of MRD, but rather reflects the current lack of direct evidence supporting its role as a validated surrogate endpoint in the context of continuous BTK inhibitor therapy.

Similarly, the SEQUOIA trial evaluated first‐line zanubrutinib monotherapy versus bendamustine–rituximab in patients with treatment‐naïve CLL, demonstrating significantly improved PFS and overall response rates with zanubrutinib [48]. Although the study was not powered to assess MRD as a primary endpoint, exploratory analyses indicated that achievement of uMRD was associated with deeper remissions in this context.

### 3.3 MRD as a Prognostic Marker in Fixed‐Duration Targeted Therapy

The introduction of venetoclax revolutionized CLL therapy, enabling finite‐duration, deep molecular remissions. In the CLL14 trial, the secondary key endpoint included the rate of uMRD (cutoff 10–4) in PB and BM, by ASO‐PCR, while exploratory endpoints included uMRD rates with the cutoffs of 10–4, 10–5, and 10–6, by NGS. The study demonstrated that venetoclax–obinutuzumab (Ven‐Obi) achieved uMRD in 75% (blood) and 57% (marrow) of patients versus 35% and 17% with chlorambucil–obinutuzumab, with uMRD strongly predicting PFS (HR 0.35) and durable remissions after treatment cessation [19, 20, 49, 50]. Interestingly, no substantial difference in uMRD rates was observed between unfit (80.3%) and fit (85.1%) patients treated with Ven‐Obi, with both groups achieving uMRD < 10^−4^ at comparable frequencies [51]. The MURANO trial confirmed fixed‐duration venetoclax–rituximab (VenR) as highly effective in relapsed/refractory CLL. MRD was centrally measured in PB using ASO‐PCR and/or MFC. Achieving uMRD at the end of treatment (EOT) predicted prolonged PFS and OS [16, 52, 53]. uMRD persistence for up to 3 years and a 25‐month lag from MRD conversion to progression underscored its predictive power. Even high‐risk genomic subgroups benefitted from achieving uMRD, confirming its universal prognostic value.

The VERITAS (GIMEMA LLC1518) [54] phase II trial demonstrated that fixed‐duration venetoclax–rituximab (VenR; 12 cycles) can achieve deep and durable molecular remissions even in young, high‐risk CLL patients. The study enrolled 75 fit patients aged ≤ 65 years, 96% with unmutated IGHV and 12% with TP53 disruption, representing a population typically considered less likely to achieve sustained MRD clearance. MRD was assessed by flow cytometry with a sensitivity of 10^−4^ and ASO‐PCR with a sensitivity of 10^−5^ in both PB and BM. By the end of treatment, uMRD was achieved in 69.3% of PB and 58.7% of BM samples—rates comparable to or exceeding those observed in the CLL14 trial, despite the higher‐risk features of the VERITAS cohort. Importantly, none of the patients who achieved uMRD in both compartments experienced disease progression after a median follow‐up of 20.8 months, and 12‐month MRD‐free survival was 73.1% among those with PB uMRD. These findings support the ability of venetoclax‐based therapy to induce deep and durable responses even in biologically adverse subgroups. From an expert perspective, the VERITAS results reinforce the role of MRD as a strong prognostic biomarker in CLL, as achieving and maintaining uMRD was associated with sustained remission. However, while the study highlights the effectiveness of a fixed‐duration approach, it did not evaluate an MRD‐guided treatment strategy. Rather than demonstrating the feasibility of MRD‐driven therapy, these data suggest that deep molecular responses can be achieved with predefined treatment duration, supporting the broader concept of time‐limited therapy in younger, high‐risk patients. If confirmed with longer follow‐up, these results may contribute to shifting treatment goals in CLL toward deep remission and treatment‐free intervals, with MRD serving as an important marker of response and disease control. The GLOW trial validated fixed‐duration ibrutinib–venetoclax (I + V) as a standard for older or comorbid patients [55, 56, 57]. Deep uMRD (< 10^−5^) was achieved in 43% (blood) and 41% (marrow) of I + V‐treated patients, with 80% maintaining uMRD for ≥ 1 year after therapy. While uMRD correlated with durable control, the small PFS gap between MRD‐negative and ‐positive patients suggests potent targeted therapy may mitigate MRD's short‐term prognostic weight.

The AMPLIFY trial confirmed the efficacy of acalabrutinib–venetoclax, with or without obinutuzumab, versus chemoimmunotherapy in untreated, fit CLL patients [22]. After a median follow‐up of 40.8 months, estimated 36‐month PFS was 76.5% with acalabrutinib–venetoclax, 83.1% with acalabrutinib–venetoclax–obinutuzumab, and 66.5% with chemoimmunotherapy. Estimated 36‐month OS was 94.1% with acalabrutinib–venetoclax, 87.7% with acalabrutinib–venetoclax–obinutuzumab, and 85.9% with chemoimmunotherapy. However, MRD analysis was limited to the < 10^−4^ threshold, providing an incomplete view of remission depth. Deeper MRD evaluation (< 10^−5^ or < 10^−6^) is needed to assess long‐term treatment‐free control.

From a biological standpoint, the addition of anti‐CD20 antibodies may enhance depth of response through complementary mechanisms, including immune‐mediated cytotoxicity (ADCC, CDC) and improved clearance of circulating and nodal disease. The question of whether triplet regimens provide a true additive or synergistic advantage over doublet combinations must also be balanced against increased toxicity, particularly infections and cardiovascular events, as well as treatment complexity. Similarly, MRD‐driven strategies have been explored in large randomized trials such as CLL13 [17], which compared chemoimmunotherapy with venetoclax‐based combinations, including triplet regimens incorporating anti‐CD20 antibodies. These studies confirmed that deeper MRD responses are achievable with combination strategies; however, they also highlighted that achieving uMRD does not necessarily require treatment intensification in all patients.

The FLAIR trial, incorporating MRD‐guided therapy, assessed by flow cytometry (10^−4^), demonstrated that two‐thirds of patients receiving ibrutinib–venetoclax achieved uMRD in BM within 2 years, compared with 48% with FCR and none with ibrutinib monotherapy. PB uMRD rates paralleled these observations (73.1%, 60.8%, and 0%, respectively), corroborating the combination's ability to achieve profound molecular remission, even among patients with high‐risk disease [58]. Over 60% of patients in the combination arm were able to discontinue therapy based on MRD, with only 5% requiring retreatment due to MRD recurrence. In contrast, MRD recurrence after FCR was substantially higher, highlighting the durability of molecular remission with targeted therapy. These molecular results translated into meaningful clinical outcomes: 5‐year PFS was 93.9% with ibrutinib–venetoclax, 79.0% with ibrutinib, and 58.1% with FCR. Benefits were particularly pronounced in unmutated IGHV patients, and overall survival also favored the combination across risk strata.

The phase II CAPTIVATE study evaluated a chemotherapy‐free regimen of ibrutinib plus venetoclax in previously untreated patients with CLL, including an MRD‐guided cohort. After an initial induction phase, patients who achieved uMRD were randomized to either treatment discontinuation (placebo) or continued ibrutinib, while MRD‐positive patients continued active therapy [59]. The study demonstrated high rates of uMRD in both PB and BM. Importantly, patients with uMRD who discontinued therapy maintained PFS rates comparable to those who continued treatment, supporting the feasibility of MRD‐guided, fixed‐duration strategies [59].

These findings highlight the role of MRD as a tool to individualize treatment duration in CLL, enabling safe treatment discontinuation in patients achieving deep responses without compromising clinical outcomes. Durable MRD negativity is more likely in patients with mutated IGHV genes, intact TP53 function, and robust immune recovery, whereas TP53 aberrations as well as an unmutated IGHV gene status increase the risk of early MRD recurrence. By integrating these biological and clinical factors with MRD kinetics, clinicians can safely individualize TFO, bridging historical chemoimmunotherapy with modern fixed‐duration targeted regimens and establishing MRD as a cornerstone of precision medicine in CLL. Ongoing trials such as MAJIC (NCT05057494) and VERITAS (NCT04285567) are exploring MRD‐adapted strategies to further individualize therapy.

At this point, it is important to distinguish between two different, although related, uses of MRD in CLL. At the trial level, MRD serves primarily as a marker of depth of remission and as a comparative endpoint to estimate the relative efficacy of regimen A versus regimen B. In this setting, a higher end‐of‐treatment uMRD rate may support the conclusion that one treatment induces deeper remissions than another. However, this does not automatically translate into the same prognostic meaning for every individual patient. At the patient level, the clinical significance of achieving uMRD depends on the treatment context, disease biology, and host‐related features.

A pooled analysis of over 8000 patients across 16 venetoclax‐based trials confirmed that MRD negativity at the end of treatment is a strong predictor of long‐term PFS and OS within these venetoclax‐based, time‐limited regimens [60]. Beyond these phase III studies, several phase II trials have prospectively incorporated MRD status into treatment‐adaptation strategies. Examples include MRD‐guided discontinuation or continuation of venetoclax‐based regimens (such as venetoclax–obinutuzumab or ibrutinib–venetoclax), whereby patients achieving uMRD stop therapy while those with persistent MRD continue or intensify treatment [39, 58]. These studies have reported encouraging uMRD rates and PFS, suggesting that MRD‐adapted approaches are feasible in closely monitored trial settings; however, they remain investigational and require confirmation in larger, randomized studies before being adopted in routine practice. Importantly, to date, no study has conclusively demonstrated that a uMRD‐directed treatment strategy is superior—in terms of survival outcomes, tolerability, or cost‐effectiveness—to a fixed‐duration approach. While trials such as FLAIR [58] have shown the superiority of MRD‐guided ibrutinib–venetoclax over chemoimmunotherapy (FCR), they did not include a direct comparison with a fixed‐duration ibrutinib–venetoclax regimen. Therefore, dedicated randomized studies directly comparing MRD‐driven versus fixed‐duration strategies are still required to establish whether MRD‐guided treatment duration translates into improved long‐term outcomes.

While the addition of anti‐CD20 antibody obinutuzumab or Bruton's tyrosine kinase BTK inhibitors to venetoclax significantly improves the rate of uMRD, a subset of patients still exhibits detectable disease after 1 or 2 years of therapy. This observation has driven interest in combining all three agents—venetoclax, a BTK inhibitor, and the CD20 monoclonal antibody, obinutuzumab—in frontline or relapsed/refractory settings to further deepen responses. Phase II studies evaluating triplet regimens have reported high rates of uMRD assessed primarily by flow cytometry (10^−4^), with some studies incorporating NGS (≤ 10^−5^). At Ohio State University, previously untreated patients achieved uMRD in BM in 67% of cases, while relapsed/refractory patients reached 50%, although PB MRD was not reported [61]. Similarly, the CLL2‐GIVe trial, focusing on treatment‐naïve patients with TP53 aberrations, reported 81% uMRD in PB, highlighting the capacity of triple therapy to induce deep molecular remission even in high‐risk populations [62]. Other studies, including the phase 2 AVO trial evaluating acalabrutinib, venetoclax, and obinutuzumab [63], and the BOVen study testing zanubrutinib‐based combinations [64], found uMRD rates in PB of 82% and 92%, respectively, after approximately 1 year of treatment.

Overall, triplet strategies demonstrate that combining a BTK inhibitor with venetoclax and obinutuzumab can determine the achievement of exceptional deep molecular remissions. Nevertheless, whether the incremental benefit of triplet therapy over doublets alone justifies the added toxicity, particularly cardiovascular adverse events and infections, remains an open question. Future randomized trials and longer follow‐up will be crucial to determine whether these deeper responses translate into superior PFS, and to define in which patient populations such intensified approaches are truly warranted.

Table 3 summarizes key studies demonstrating the prognostic and clinical value of MRD in CLL [2, 10, 16, 19, 45, 46, 49, 53, 54, 56, 57, 58, 65, 66].

**TABLE 3:** MRD outcomes and progression‐free survival in key CLL trials.

Trial	Treatment	Population	MRD assessment	uMRD rate (bone marrow, %)	uMRD rate (peripheral blood, %)	5‐year PFS (%)	Notes
FLAIR [58]	Ibrutinib monotherapy	Treatment‐naive	Flow (10^−4^)	< 5	< 5	79.0	Continuous therapy; no patients achieved uMRD
FLAIR [58]	Ibrutinib–venetoclax	Treatment‐naive	Flow (10^−4^)	66.2	73.1	93.9	MRD‐guided discontinuation; 5% required retreatment
FLAIR [58]	FCR	Treatment‐naive	Flow (10^−4^)	48.3	60.8	58.1	Standard 6‐cycle chemoimmunotherapy; MRD recurrence In > 50% of uMRD patients
CLL8 [45]	FCR	Treatment‐naive	Flow (10^−4^) ASO‐PCR (10^−5^)	44	50–60	60–65	Older pivotal GCLLSG trial; uMRD strongly predicted PFS and OS
CLL11 [65]	Chlorambucil–obinutuzumab	Treatment‐naive	ASO‐PCR (10^−4^)	17	35	—	First‐line in older/comorbid patients; lower MRD and PFS versus Ven‐Obi
CLL14 [19, 49, 50]	Venetoclax–obinutuzumab	Treatment‐naive	Flow (10^−4^) NGS (≤ 10^−5^)	57	75	—	Fixed‐duration; uMRD strongly predicted PFS
MURANO [16, 53]	Venetoclax–rituximab	Relapsed/refractory	ASO‐PCR (10^−4^)	60	60–70	—	End‐of‐treatment uMRD predicted prolonged PFS and OS
GLOW [56, 57]	Ibrutinib–venetoclax	Treatment‐naive	Flow (10^−4^) NGS (≤ 10^−5^)	55–60	77	—	Fixed‐duration; 80% maintained uMRD ≥ 1 year
SEQUOIA [48]	Zanubrutinib	Treatment‐naive	Flow (10^−4^) NGS (≤ 10^−5^)	< 5	< 5	—	First‐line; Exploratory MRD analysis suggested deeper remissions than BR
BR (various trials) [2]	Bendamustine–rituximab	Treatment‐naive	Flow (10^−4^) NGS (≤ 10^−5^)	10–25	15–30	40–55	Lower uMRD and PFS compared to FCR; used in older/frail patients
VERITAS (LLC1518) [54]	Venetoclax–rituximab	Relapsed/refractory	Flow (10^−4^)	58.7	69.3	—	Young, fit, high‐risk patients; uMRD strongly associated with durable remissions
GAIA/CLL13 [17, 66]	Venetoclax–obinutuzumab	Treatment‐naive	NGS (≤ 10^−5^) Flow (10^−4^)	87	87	88 (3‐year)	Fit, previously untreated patients; high uMRD rates; fixed‐duration therapy
GAIA/CLL13 [17, 66]	Venetoclax–obinutuzumab–ibrutinib	Treatment‐naive	NGS (≤ 10^−5^)	92	92	91 (3‐year)	Triple therapy; marginal uMRD improvement versus Ven‐Obi; similar PFS; time‐limited regimen

## 4 Future Perspectives

A meta‐analysis demonstrates that venetoclax‐based combinations, particularly venetoclax–obinutuzumab (Ven‐Obi), achieve the deepest molecular remissions among first‐line therapies, with the highest rates of uMRD in both BM and PB. In contrast, BTK inhibitor monotherapies such as ibrutinib seldom achieve MRD negativity, underscoring the superior depth of response with time‐limited Bcl‐2 inhibitor–based regimens [67]. However, the study also underscores ongoing challenges—most notably the lack of uniform MRD assessment methodologies across trials, which complicates cross‐study comparison. Furthermore, while uMRD status predicts favorable outcomes, its role in guiding treatment cessation in fit, high‐risk populations requires prospective validation, particularly in the context of triplet regimens incorporating BTK inhibitors, Bcl‐2 inhibitors, and CD20 antibodies.

The future of MRD‐guided therapy in CLL lies in integrating traditional molecular monitoring with emerging technologies such as ctDNA analysis and AI. ctDNA, shed from leukemic cells into the bloodstream, offers a complementary approach to conventional MRD assessment, particularly in capturing spatially heterogeneous disease or extramedullary involvement that may escape detection by BM or PB sampling. Pilot studies have shown that ctDNA‐based MRD assessment can detect impending relapse earlier than standard multiparameter flow cytometry and may provide a theoretical window for preemptive intervention within clinical trials. However, it should be emphasized that there is currently no evidence that earlier treatment based on MRD or ctDNA detection improves overall survival in CLL. Moreover, such strategies may carry potential risks, including overtreatment, immunosuppression, toxicity, increased costs, and clonal selection, and therefore remain investigational [68]. This early‐warning capability is particularly relevant for patients undergoing time‐limited therapy, where the timing of MRD reappearance can guide retreatment and prolong remission within the framework of clinical trials and carefully designed monitoring protocols.

In the near future, AI may contribute to advancing CLL management by integrating complex datasets, including MRD kinetics, genomic profiles, immunophenotypic recovery, and patient‐specific clinical variables. Early studies suggest that AI‐based models can identify patterns associated with relapse risk or durable remission, raising the possibility of more personalized approaches to treatment duration. For example, machine‐learning algorithms trained on longitudinal MRD measurements, combined with mutational burden and immune reconstitution parameters, have shown potential in estimating the probability of sustained MRD negativity, which could eventually support clinical decision‐making regarding treatment continuation, maintenance strategies, or TFO [69]. However, these approaches remain investigational and are not currently used to guide routine clinical practice. While AI‐driven predictive models may, in the future, refine risk stratification beyond conventional static thresholds, their clinical utility requires prospective validation. At present, they should be viewed as promising research tools rather than established decision‐support systems for individualized, adaptive therapy strategies [69].

From a regulatory perspective, both the US Food and Drug Administration (FDA) and the European Medicines Agency (EMA) are actively evaluating MRD as a validated surrogate endpoint in CLL trials. Recognition of MRD as an endpoint could accelerate drug approvals and foster the development of adaptive, response‐driven trial designs, where therapy duration or intensity is modulated based on molecular response. Ongoing clinical studies, including VERITAS (NCT04285567) and AMPLIFY (NCT05209074), aim to define clinically actionable MRD thresholds for therapy cessation, while real‐world registries such as ERIC‐MRDnet will provide external validation of MRD‐guided approaches across broader, more heterogeneous patient populations.

The integration of ctDNA monitoring, AI‐driven analytics, and MRD assessment has the potential to drive a major evolution in the management of CLL. By facilitating earlier detection of molecular relapse, enabling precise personalization of therapy duration, and allowing real‐time synthesis of complex biological data, these advances may enhance therapeutic efficacy, reduce treatment‐related toxicity, and broaden the applicability of treatment‐free remission strategies if validated in prospective studies.

Ultimately, the incorporation of AI into MRD‐guided treatment paradigms may, in the long term, contribute to a more dynamic, patient‐specific model of care in which therapy duration and intensity are more closely aligned with individual molecular risk, rather than being fixed a priori.

## 5 Conclusion

The integration of MRD into CLL management has fundamentally advanced precision oncology, establishing MRD as a robust biomarker that predicts PFS and OS across treatment modalities and, in particular, within venetoclax‐based time‐limited regimens and chemoimmunotherapy. Achieving uMRD has become an important therapeutic goal in many trials and an indicator of a favorable prognosis, which in selected venetoclax‐based studies has been associated with the safe implementation of TFO, reducing cumulative toxicity, preserving quality of life. Data from trials such as CAPTIVATE and FLAIR support the feasibility of MRD‐adapted treatment discontinuation in well‐defined settings [58, 59], but longer follow‐up and additional studies are required before such strategies can be generalized to routine practice.

Despite this progress, operational and systemic challenges limit widespread adoption. Assay heterogeneity—across MFC, ASO‐PCR, and NGS—can produce variable results, complicating comparability between centers and clinical trials. Standardization efforts, such as ERIC and EuroFlow protocols and validated NGS platforms like clonoSEQ, are essential to ensure reproducibility and reliable interpretation. Cost and reimbursement barriers further constrain routine MRD assessment, although economic analyses indicate that MRD‐guided therapy can reduce overall healthcare expenditures by shortening treatment duration and decreasing drug‐related toxicity.

Implementation barriers remain, including clinician uncertainty in interpreting low‐level MRD positivity. Overcoming this requires guideline incorporation (NCCN, ESMO) and educational initiatives to empower clinicians with standardized, evidence‐based approaches. Complementary technologies, including the ctDNA monitoring and AI‐driven predictive modeling, promise to enhance MRD‐guided precision by capturing disease heterogeneity, refining risk stratification, and personalizing therapy duration.

Regulatory recognition of MRD as a surrogate endpoint by the FDA and EMA is likely to accelerate adaptive trial designs, support drug approvals, and embed MRD assessment into research and, potentially, future practice. Future research will focus on defining clinically actionable MRD thresholds. Indeed, while MRD negativity is conventionally defined using a threshold of 10^−4^, increasing assay sensitivity has enabled the identification of deeper levels of undetectable disease (e.g., 10^−5^ and 10^−6^), raising the question of whether progressively deeper responses translate into incremental clinical benefit. In this contest several studies, particularly those employing NGS‐based approaches, suggest that achieving MRD negativity below 10^−5^ or even 10^−6^ is associated with longer PFS compared to patients with MRD levels detectable at 10^−4^ [40]. Moreover, future studies will optimize monitoring intervals and validate MRD‐guided strategies across diverse patient populations, moving closer to the goal of maximally durable treatment‐free remission.

Ultimately, MRD currently represents a central prognostic and research tool in CLL. With further validation, MRD‐adapted strategies may support more adaptive, patient‐specific management, enabling response‐guided care that aims to maximize efficacy while minimizing toxicity.

In conclusion, MRD quantification is reshaping the clinical and regulatory landscape in CLL, moving the field toward precision‐based, response‐adapted therapy. Briefly, an MRD negativity in the setting of time‐limited regimens may identify those cases that will experience a durable response, while for patients receiving continuous therapy, although less common, it may help individualize candidates for treatment discontinuation within clinical trials or highly selected scenarios. Moreover, MRD assessment in routine practice has the potential to predict clinical relapse, providing a useful tool for clinicians, guiding patients' follow‐up and, possibly, their enrollment within clinical trials for preventive therapy. Finally, MRD may help tailor therapy intensity. Future trials integrating standardized MRD monitoring, real‐world validation, and long‐term therapy‐free remission endpoints will be critical to fully establish whether and how MRD‐guided therapy should be incorporated as a component of modern CLL management.

## Author Contributions

All authors contributed to the manuscript and were involved in revisions and proofreading. All authors approved the submitted version.

## Funding

This work was supported by PNRR, PNRR‐MAD‐2022‐12375673.

## Ethics Statement

The authors have nothing to report.

## Conflicts of Interest

The authors declare no conflicts of interest.

## Data Availability

Data sharing not applicable to this article as no datasets were generated or analyzed during the current study.
